# Urinary Calcium Measurement in Patients With Hypercalcaemia; Endocrine Physicians and Surgeons Survey Results From UK

**DOI:** 10.1111/cen.70008

**Published:** 2025-08-03

**Authors:** Muhammad Fahad Arshad, Saba P. Balasubramanian

**Affiliations:** ^1^ Sheffield Teaching Hospitals NHS Foundation Trust Sheffield UK; ^2^ University of Sheffield Sheffield UK

**Keywords:** hypercalcaemia, survey, urinary calcium

## Abstract

**Background:**

In patients with hypercalcaemia, assessment of urinary calcium excretion helps differentiate primary hyperparathyroidism (PHPT) from familial hypocalciuric hypercalcaemia (FHH). For this, 24 h calcium to creatinine clearance ratio (CCCR) is recommended, but others tests like random CCCR, 24 h urine calcium excretion (UCE), and calcium to creatinine ratio (CR) are also frequently used.

**Objective:**

The survey objective was to evaluate current practice among UK endocrinologists and surgeons.

**Methods:**

A web‐based anonymous cross‐sectional survey, consisting of eight multiple‐choice questions was developed using Survey Monkey. The survey was disseminated to members of British Association of Endocrine and Thyroid Surgeons (BAETS) and Society for Endocrinology (SfE) between November 20, 2025 and December 19, 2024.

**Results:**

Two hundred and sixty‐six responses from 210 endocrinologists and 56 surgeons were received (85% consultants). Respondents worked in both university (48.9%) and district hospitals (47.7%). The most commonly performed urine calcium test in hypercalcaemic patients was 24 h UCE (58.6%), but for PHPT versus FHH differentiation, the most preferred test was 24 h CCCR (43.6%), followed by random CCCR (24.8%), 24 h UCE (14.3%), and CR (16.5%). Of respondents who had experience with using CCCR (*n* = 235), most (55.6%) used a cut‐off of > 0.01 to rule out FHH, while > 0.02 cut off was used by 26.7% respondents. Most clinicians (70.3%) used albumin‐adjusted calcium for CCCR calculation, and 71.4% respondents considered vitamin D levels ≥ 50 nmol/L to be adequate for urinary calcium measurement.

**Conclusion:**

The survey provides valuable insight into current UK practice. 24 h and random CCCR are the most commonly used tests to exclude FHH, but overall, practice varies widely.

## Conflicts of Interest

The authors declare no conflicts of interest.

To the Editor,

Primary hyperparathyroidism (PHPT) is the third most common endocrine condition after diabetes mellitus and hypothyroidism, with an estimated prevalence of around 1% [1]. The biochemistry in mild to moderate PHPT overlaps with familial hypocalciuric hypercalcaemia (FHH), an autosomal dominant condition characterised by CASR, APS21, or GNA11 gene mutations [1, 2]. FHH is much rarer than PHPT, but it is vital to exclude this condition before proceeding to surgery in PHPT [2]. Urinary calcium studies are widely used to exclude FHH and avoid unnecessary surgery [1, 3].

The 24 h calcium to creatinine clearance ratio (CCCR) is considered the test with high specificity to exclude FHH and therefore, recommended by the fifth International workshop on PHPT [1, 2]. However, the National Institute for Health and Care Excellence (NICE) in the United Kingdom (UK) recommends 24 h urinary calcium excretion (UCE), or random CCCR, or random calcium to creatinine ratio (CR) to differentiate PHPT from FHH [4]. No thresholds were recommended by NICE for these tests. In clinical practice, however, there is significant heterogeneity [3].

We developed a web‐based cross‐sectional survey for endocrinologists and endocrine surgeons in the United Kingdom to understand the current practice on urinary calcium testing in patients with hypercalcaemia. The survey consisted of eight multiple‐choice questions on (i) speciality, grade, and type of hospital of practice, (ii) preferred type of urinary calcium measurements and thresholds, (iii) vitamin D status threshold and use of urinary sodium measurement (Supporting Information file). The survey was disseminated to all members of British Association of Endocrine and Thyroid Surgeons (BAETS) (*n* = 445 UK members) and Society for Endocrinology (SfE) (*n* = 1609 UK clinicians) via email. The responses were collected anonymously using an online survey platform (Survey Monkey) between November 20 and December 19, 2024. The survey was approved by the BAETS executive committee and the SfE Endocrine Specialist Network (ESN) on bone and calcium for distribution.

We received a total of 266 responses of which 56 responses were from surgeons and 210 responses were from endocrinologists (response rate 12.6% and 13.1%). The results of the survey are summarised in Table 1. Most respondents (85.0%) were working at consultant level (surgeons 98%; endocrinologists 81%) and distributed almost evenly among university (48.9%) and district general (47.7%) hospitals.

**Table 1:** Summary of survey results.

	Surgery (*n* = 56)	Endocrinology (*n* = 210)	Total (*n* = 266)
Main clinical role *n* (%)	Consultants = 55 (98.2%) Registrars = 1 (1.8%) Others = 0	Consultants = 171 (81.4%) Registrars = 31 (14.8%) Others = 8 (3.8%)	Consultants = 226 (85.0%) Registrars = 32 (12.0%) Others = 8 (3.0%)
Place of work *n* (%)	District general/secondary hospital = 21 (37.5%) University/tertiary hospital = 34 (60.7%) Others = 1 (1.8%)	District general/secondary hospital = 106 (50.5%) University/tertiary hospital = 96 (45.7%) Others = 8 (3.8%)	District general/secondary hospital = 127 (47.7%) University/tertiary hospital = 130 (48.9%) Others = 9 (3.4%)
Tests done in hypercalcaemic patients *n* (%) Note: selection of multiple options allowed for this question	24 h UCE = 35(62.5%) 24 h CCCR = 25 (44.6%) Random CCCR = 18 (32.1%) CR ratio = 12 (21.4%) Others/unsure/do not check urine calcium = 11 (19.6%)	24 h UCE = 121 (57.6%) 24 h CCCR = 97 (46.2%) Random CCCR = 76 (36.2%) CR ratio = 32 (15.2%) Others/unsure/do not check urine calcium = 20 (9.5%)	24 h UCE = 156 (58.6%) 24 h CCCR = 122 (45.9%) Random CCCR = 94 (35.3%) CR ratio = 44 (16.5%) Others/unsure/do not check urine calcium = 31 (11.7%)
Most commonly used test for PHPT versus FHH *n* (%)	24 h CCCR = 25 (44.6%) Random CCCR = 10 (17.9%) 24 h UCE = 8 (14.3%) CR ratio = 6 (10.7%) Others/unsure = 7 (12.5%)	24 h CCCR = 91 (43.3%) Random CCCR = 56 (26.7%) 24 h UCE = 30 (14.3%) CR ratio = 17 (8.1%) Others/unsure = 16 (7.6%)	24 h CCCR = 116 (43.6%) Random CCCR = 66 (24.8%) 24 h UCE = 38 (14.3%) CR ratio = 23 (8.6%) Others/unsure = 23 (8.6%)
Cut off to exclude FHH for CCCR (*n* = 251) *n* (%)	> 0.01 = 32 (61.6%) > 0.02 = 11 (21.1%) Others/unsure = 9 (17.3%)	> 0.01 = 116 (58.3%) > 0.02 = 60 (30.1%) Others/unsure = 23 (11.6%)	> 0.01 = 148 (59.0%) > 0.02 = 71 (28.3%) Others/unsure = 47 (18.7%)
Type of calcium measurement for CCCR (*n* = 232) *n* (%)	Adjusted calcium = 36 (64.3%) Unadjusted calcium = 2 (3.6%) Unsure = 18 (32.1%)	Adjusted calcium = 151 (71.9%) Unadjusted calcium = 20 (9.5%) Unsure = 39 (18.6%)	Adjusted calcium = 187 (70.3%) Unadjusted calcium = 22 (8.3%) Unsure = 57 (21.4%)
Adequate vitamin D level for urine calcium measurement *n* (%)	≥ 50 nmol/L = 30 (53.6%) ≥ 75 nmol/L = 6 (10.7%) ≥ 25 nmol/L = 6 (10.7%) Others/do not measure/unsure = 14 (25%)	≥ 50 nmol/L = 160 (76.2%) ≥ 75 nmol/L = 17 (8.1%) ≥ 25 nmol/L = 13 (6.2%) Others/Do not measure/unsure = 20 (9.5%)	≥ 50 nmol/L = 190 (71.4%) ≥ 75 nmol/L = 23 (8.6%) ≥ 25 nmol/L = 19 (7.1%) Others/do not measure/unsure = 35 (12.8%)
Simultaneous urinary sodium measurement *n* (%)	Never < 1% = 22 (39.3%) Rarely (1%−20%) = 9 (16.1%) Sometimes (20%−50%) = 1 (1.8%) Mostly (50%−80%) = 2 (3.6%) Always (> 80%) = 8 (14.3%) Unsure/don't check 24 h urine calcium = 16 (28.6%)	Never < 1% = 137 (65.2%) Rarely (1%−20%) = 24 (11.4%) Sometimes (20%−50%) = 11 (5.2%) Mostly (50%−80%) = 3 (1.4%) Always (> 80%) = 6 (2.8%) Unsure/don't check 24 h urine calcium = 29 (13.8%)	Never < 1% = 159 (59.8%) Rarely (1%−20%) = 33 (12.4%) Sometimes (20%−50%) = 12 (4.5%) Mostly (50%−80%) = 5 (1.9%) Always (> 80%) = 14 (5.3%) Unsure/don't check 24 h urine calcium = 43 (16.2%)

Abbreviations: CCCR = calcium to creatinine clearance ratio, FHH = familial hypocalciuric hypocalcemia, PHPT = primary hyperparathyroidism, UCE = urine calcium excretion.

In hypercalcaemic patients, most clinicians would perform 24 h UCE, followed by 24 h CCCR, random CCCR, CR, and fasting CCCR, respectively. To differentiate FHH from PHPT, the most commonly performed test was 24 h CCCR (43.6%), followed by random CCCR (24.8%), 24 h UCE (14.3%), and CR (8.6%). Of the respondents who had experience using CCCR (*n* = 251), a > 0.01 cut off was used by most (55.6%) to exclude FHH from PHPT. 26.7% respondents used a cut‐off of > 0.02 to rule out FHH in patients with hypercalcaemia, while 17.7% clinicians were either unsure or relied on other options such as outcome of the multidisciplinary meeting, clinical context, and so forth.

When using any type of CCCR, 70.3% clinicians (*n* = 187) used albumin‐adjusted calcium concentration for CCCR calculation. Most clinicians (71.4%) considered vitamin D levels on ≥ 50 nmol/L to be adequate for any urinary calcium measurement. Simultaneous urinary sodium measurement was never or rarely undertaken by most clinicians (72.2%).

Our survey also shows that in line with international guidelines, 24 h CCCR was the most preferred test to differentiate PHPT from FHH. However, 1/4th of the clinicians opted for random CCCR. This is very plausibly due to the convenience of the test as 24 h urine collections are more demanding, especially for older patients. In another recent survey of 50 clinicians in the United Kingdom, similar results were noted [3], where 54% of the clinicians selected 24 h CCCR, while spot (random, morning, or fasting) testing was first choice for 32% of the survey respondents [3]. In another report from one centre in the United Kingdom, there was a clear preference for random CCCR as the first test in the diagnostic work‐up [5] demonstrating a strong inclination towards random testing in at least some centres. Nonetheless, it should be noted here that the accuracy of a random urine test in excluding FHH has not yet been formally studied [3]. The only studies evaluating the spot urinary calcium tests have been performed in patients with kidney stones without hypercalcaemia and the results are not always consistent [3]. This survey showed some variation in how CCCR (24 h or random) results were interpreted. Most clinicians (56%) were reassured by > 0.01 cut off to exclude FHH but others (27%) selected a higher > 0.02 cut off. This variation can be somewhat explained as up to 80% of patients with FHH can have CCCR of < 0.01 [2]. Moreover, several PHPT patients can also have a low CCCR indicating a considerable overlap in urinary calcium excretion among the two conditions.

Despite choosing CCCR (24 h or random) for excluding FHH in hypercalcaemic patients, it is interesting to note that 24 h UCE was still the most performed test in these patients. The survey did not explore the reason for this trend, but it is likely on the basis that hypercalciuria is considered a high‐risk for development of renal stones and therefore an indication for surgery [2]. Most clinicians checked vitamin D levels for urine calcium testing and considered a cut off of 50 nmol/L as satisfactory. This was also comparable (61%) in the other UK survey mentioned above [3]. An explanation for this preference could be that this threshold is the lower limit of the normal reference range adopted by most UK centres.

This survey has several limitations. While the aim of this survey was to focus on the urinary calcium testing in typical patients with hypercalcaemia, other factors (clinical presentation, degree of hypercalcaemia, parathyroid hormone levels, presence of renal stones, osteoporosis etc) not studied in this survey may influence the diagnostic work‐up of hypercalcaemic patients. Like other surveys, this only reports clinicians' preferences instead of real patient data which would have provided stronger evidence for the real‐world practice. To conclude, the survey demonstrates significant heterogeneity in clinical practice across the UK. The survey indicates several gaps in research, calling for further studies to standardise patient care and promote evidence‐based practice.

## Supporting information

Survey Supplementary file.

## Data Availability

The data for this survey is summarised in the manuscript and is not publicly available. The raw data of this project is available from the corresponding author upon reasonable request.
