# Supplementary material for: Urinary Calcium Measurement in Patients With Hypercalcaemia; Endocrine Physicians and Surgeons Survey Results From UK
Source: Clin Endocrinol (Oxf). 2025 Aug 3;103(5):757–60. doi: 10.1111/cen.70008 (PMC12492780; doi:10.1111/cen.70008)
Supplement: Supplementary file 1 — Survey Supplementary file. [file CEN-103-757-s001.docx]

**Survey on Urinary Calcium measurement in patients with Hypercalcaemia**

1. **Main clinical role?**

- Endocrinology- Consultant
- Endocrinology- Registrar
- Endocrine Surgery- Consultant
- Head & Neck Surgery- Consultant
- Surgery- Registrar
- Other

1. **Place of work?**

- University Hospital / Tertiary Hospital
- University Hospital / Tertiary Hospital
- Other

1. **Which urine tests do you perform in patients with hypercalcaemia and raised PTH levels? (Select multiple tests if needed)**

- 24-hour urine calcium excretion
- Random calcium to creatinine clearance ratio (CCCR)
- Fasting calcium to creatinine clearance ratio (CCCR)
- 24-hour calcium to creatinine clearance ratio (CCCR)
- Calcium to creatinine ratio
- Overnight urine calcium excretion
- Unsure
- None
- Other

1. **Which urine tests do you primarily use in your clinical practice in hypercalcaemic patients to differentiate Primary Hyperparathyroidism (PHPT) from Familial Hypocalciuric Hypercalcaemia (FHH)? (Select one option only)**

- 24-hour urine calcium excretion
- Random calcium to creatinine clearance ratio (CCCR)
- Fasting calcium to creatinine clearance ratio (CCCR)
- 24-hour calcium to creatinine clearance ratio (CCCR)
- Calcium to creatinine ratio
- Overnight urine calcium excretion
- Unsure
- None
- Other

1. **If you use calcium to creatinine clearance ratio in your practice, then which cut-off do you use to rule out Familial Hypercalcaemic Hypocalciuria? (Select one option only)**

- >0.01
- >0.02
- Not applicable
- Unsure
- Other

1. **If you use (or interpret) urinary calcium excretion measurements (any type) in your clinical practice, which Vitamin D level would you consider appropriate for checking urinary calcium? (Select one option only)**

- ≥ 25 nmol/L or equivalent
- ≥ 50 nmol/L or equivalent
- ≥ 75 nmol/L or equivalent
- Do not routinely measure vitamin D levels
- Unsure

1. **If you assess 24-hour urine calcium excretion, do you check urine sodium excretion simultaneously? (Select one option only)**

- Always (>95%)
- Almost always (80-95%)
- Mostly (50-80%)
- Sometimes (20-50%)
- Rarely (1-20%)
- Never (<1%)
- Unsure
- Do not measure 24-hour urine calcium

1. **If you assess calcium to creatinine clearance ratio (any type), which of the following calcium results you use (or your lab does) to calculate the ratio? (Select one option only)**

- Total (unadjusted calcium)
- Adjusted calcium
- Unsure
- Do not check calcium to creatinine clearance ratio

1. **If you would like to add any additional comments, please add these here.**

_______________________________________________________________________________________________________________________________________________________________________________________________________________________________________________________________________________________________________________
